# VAMP-2, SNAP-25A/B and syntaxin-1 in glutamatergic and GABAergic synapses of the rat cerebellar cortex

**DOI:** 10.1186/1471-2202-12-118

**Published:** 2011-11-17

**Authors:** Vincenzo Benagiano, Loredana Lorusso, Paolo Flace, Francesco Girolamo, Anna Rizzi, Lorenzo Bosco, Raffaele Cagiano, Beatrice Nico, Domenico Ribatti, Glauco Ambrosi

**Affiliations:** 1 Dipartimento Scienze Mediche di Base - Sezione di Anatomia e Istologia, Università Bari - 70124 Bari - Italy; 2 Dipartimento Bioetica, Università Bari - 70124 Bari - Italy; 3 Dipartimento Scienze Biomediche e Oncologia Umana - Sezione Farmacologia, Università Bari - 70124 Bari - Italy

## Abstract

**Background:**

The aim of this study was to assess the distribution of key SNARE proteins in glutamatergic and GABAergic synapses of the adult rat cerebellar cortex using light microscopy immunohistochemical techniques. Analysis was made of co-localizations of vGluT-1 and vGluT-2, vesicular transporters of glutamate and markers of glutamatergic synapses, or GAD, the GABA synthetic enzyme and marker of GABAergic synapses, with VAMP-2, SNAP-25A/B and syntaxin-1.

**Results:**

The examined SNARE proteins were found to be diffusely expressed in glutamatergic synapses, whereas they were rarely observed in GABAergic synapses. However, among glutamatergic synapses, subpopulations which did not contain VAMP-2, SNAP-25A/B and syntaxin-1 were detected. They included virtually all the synapses established by terminals of climbing fibres (immunoreactive for vGluT-2) and some synapses established by terminals of parallel and mossy fibres (immunoreactive for vGluT-1, and for vGluT-1 and 2, respectively). The only GABA synapses expressing the SNARE proteins studied were the synapses established by axon terminals of basket neurons.

**Conclusion:**

The present study supplies a detailed morphological description of VAMP-2, SNAP-25A/B and syntaxin-1 in the different types of glutamatergic and GABAergic synapses of the rat cerebellar cortex. The examined SNARE proteins characterize most of glutamatergic synapses and only one type of GABAergic synapses. In the subpopulations of glutamatergic and GABAergic synapses lacking the SNARE protein isoforms examined, alternative mechanisms for regulating trafficking of synaptic vesicles may be hypothesized, possibly mediated by different isoforms or homologous proteins.

## Background

According to the hypothesis of the soluble N-ethylmaleimide sensitive factor attachment receptor (SNARE), common molecular mechanisms exist in chemical synapses, that regulate the processes of docking, priming and fusion between the synaptic vesicle membrane and specific regions of the plasma membrane (target membrane) and the subsequent release (exocytosis) of neurotransmitters. In the original formulation of the SNARE hypothesis, three proteins were considered, one localized on the vesicle membrane (v-SNARE), synaptobrevin or vesicular associated membrane protein (VAMP), and two localized on the target membrane (t-SNAREs), syntaxin and synaptosomal associated protein of 25 kDa (SNAP-25) [1,2]. When a synaptic site is depolarized, calcium ions penetrate the site and trigger VAMP, syntaxin and SNAP-25 to bind together in a lock and key fashion to form a tripartite structure, the SNARE complex (or core). This serves as a receptor for the cytoplasmic ATP-ase protein, N-ethylmaleimide sensitive factor (NSF), and its soluble attachment proteins. Finally, ATP hydrolysis by NSF leads to exocytosis and induces a conformational change in the complex that results in its disassembly [3-9]. Several biochemical and immunohistochemical studies suggest the existence of SNARE mechanisms virtually in all regions of the CNS [10-15].

Multiple isoforms and homologous proteins, originated by differential splicing of single genes or codified by different genes of the same family, respectively, have been described for VAMP [16], SNAP-25 [17-21] and syntaxin [18,22-25]. The different isoforms and homologous proteins display distinct patterns of distribution within CNS synapses, being present in one type of synapse and absent in another, and it has been hypothesized that when a SNARE protein is lacking, a different isoform or homologous protein will perform its function [26-29]. Therefore, SNARE mechanisms should be present in all chemical synapses, but the expression of SNARE proteins may vary. In particular, specific sets of SNARE proteins seem to be required to regulate trafficking of vesicles containing different types of neurotransmitters, possibly even one set per neurotransmitter [30,31]. According to this hypothesis, scholars have reported that SNARE proteins are strongly expressed in excitatory (glutamatergic) synapses and may be lacking in inhibitory (GABAergic) synapses [12,15,32-34].

The cerebellar cortex features a high concentration of synapses, mostly of two main types, namely glutamatergic synapses and GABAergic synapses, each type displaying distinct patterns of distribution within the cortical layers. In the molecular layer, the glutamatergic synapses are densely distributed in the neuropil, mainly consisting of terminals of parallel fibres and, to a lesser extent, of climbing fibres on the dendritic trees of Purkinje neurons [35-40]. In the same layer, GABAergic synapses are distributed throughout the neuropil and consist mainly of synapses of stellate neuron axon terminals on dendrite trees of Purkinje neurons [41-43]. In the Purkinje neuron layer, only GABAergic synapses are present, concentrated at the deep pole of the body of Purkinje neurons and formed by terminals of basket neuron axons [42-44]. In the granular layer, glutamatergic synapses and GABAergic synapses are both located in the neuropil regions scattered among granules (islands of Held), consisting of multiple synapses (glomeruli) established by glutamatergic terminals of mossy fibres and of axons of unipolar brush neurons [35-38,45] and GABAergic axon terminals of Golgi, candelabrum and Lugaro neurons [44,46,47], on granule dendrites.

Studies have reported widespread SNARE mechanisms at cerebellar cortex synapses [11,13,34,48-54], but a detailed morphological description of the distribution of v-SNARE and t-SNARE proteins in different types of glutamatergic and GABAergic synapses of the cerebellar cortex has not yet been supplied. Owing to the different patterns of distribution of glutamatergic and GABAergic synapses in the cerebellar cortex, it seems to be an ideal region where assess the distribution of v-SNARE and t-SNARE proteins within glutamatergic synapses and GABAergic synapses. In the present study, using double labelling light microscopy immunohistochemistry techniques, we examined the cortical distribution of the co-localization of VAMP or SNAP-25 or syntaxin with markers of glutamatergic or GABAergic synapses. To reveal the SNARE proteins, we used antibodies directed against isoforms considered to be of crucial importance in the CNS synapses, namely VAMP-2 [55,56], SNAP-25A and B [11,34] and syntaxin-1 [32,57]. The glutamatergic terminals were visualized by two different antibodies directed against the glutamate vesicular transporters, vGlutT-1 and vGlutT-2. This was necessary because vGluT-1 is expressed by terminals of parallel and mossy fibres, but not those of climbing fibres, and vGluT-2 is expressed by terminals of climbing and mossy fibres, but not those parallel fibres [58-62]. The GABAergic terminals were labelled using antibodies against isoforms 65 and 67 of glutamic acid decarboxylase (GAD), the enzyme involved in GABA synthesis, which labels axon terminals of GABAergic neurons of the cerebellar cortex, providing distribution patterns comparable to those of the vesicular GABA transporter (v-GAT) [63].

## Methods

### Animals and samples

Experiments were conducted on Wistar adult rats (Harlan SRC, Milano, Italy), aged 3 months, weighting 350-450 g, and housed in accordance with the Italian Ministry of Health guidelines (D.L. 116/92 and D.L. 111/94-B), the Declaration of Helsinki and National Institute of Health guidelines for the care and use of laboratory animals.

The animals were anaesthetized with equithesin (3 ml kg^-1^, injected i.p.) and transcardially perfused with a fixative solution containing 10% formaldehyde, 1% picric acid and 1% glutaraldehyde.

Brains were removed from the skull, and cerebella were isolated, sectioned in two parts on the median sagittal plane and postfixed by immersion in the same fixative solution for 1.5 h at 4°C. Each hemicerebellum was dehydrated in an ethanol series, embedded in paraffin and serially cut into 5 μm sagittal sections.

From each series of sections of cerebellar cortex, consecutive sections were chosen at intervals of 200 μm, in order to analyze all lobules of anterior and posterior lobe of cerebellum, and subjected to the following distributional analyses using immunofluorescence techniques: i. vGluT-1 and vGluT-2; ii. vGluT-1 and VAMP-2; iii. vGluT-1 and SNAP-25A/B; iv. vGluT-1 and syntaxin-1; v. vGluT-2 and VAMP-2; vi. vGluT-2 and SNAP-25A/B; vii. vGluT-2 and syntaxin-1; viii. GAD-65/67 and VAMP-2; ix. GAD-65/67 and SNAP-25A/B; x. GAD-65/67 and syntaxin-1.

### Immunohistochemistry

#### Antibodies

The following commercial antibodies were used: 1. anti-vGluT-1 polyclonal antibody, raised in guinea pig against a synthetic linear peptide from rat vGluT-1 (Millipore, Billerica, MA, USA) [34,64]; 2. anti-vGluT-2 polyclonal antibody, raised in rabbit against a strep-tag fusion protein of rat vGluT-2 (Synaptic System, Goettingen, Germany) [34,65]; 3. (a) anti-GAD monoclonal antibody, raised in mouse against human GAD-65-GST fusion protein (Stressgen, Victoria, BC, Canada), and (b) anti-GAD polyclonal antibody, raised in rabbit against a synthetic peptide corresponding to the rat GAD-65 C-terminus residues 572-585 (Chemicon), which react to both GAD-65 and GAD-67 [63]; 4. (a) anti-VAMP monoclonal antibody, raised in mouse against crude synaptic immunoprecipitate (human) (Millipore), and (b) anti-VAMP polyclonal antibody, raised in rabbit against a synthetic peptide based on rat VAMP-2 (Stressgen), which react to VAMP-2 [48]; 5. anti-SNAP-25 monoclonal antibody, raised in against crude synaptic immunoprecipitate (human), which reacts with both SNAP-25A and SNAP-25B (Chemicon) [15]; 6. anti-syntaxin monoclonal antibody, raised in mouse against clone HCP-1, which reacts to syntaxin-1 (ABCAM, Cambridge, UK) [57].

#### Immunolabelling procedures

The sections were deparaffinized, rehydrated and immunolabelled as follows. 1. Immersion in sodium citrate buffer (0.01M, pH 6.0) for 3 × 5 min in microwave oven at 700 W, to unmask antigens. 2. Treatment for 60 min at room temperature (RT) with a blocking solution containing 1% bovine serum albumin, 5% fetal calf serum and 10% normal serum in sodium phosphate buffer (PBS) (0.01M pH 7.6), to suppress non-specific immunolabelling. 3. Incubations overnight at 4°C with the primary antibodies at the following dilutions in blocking solution: (a) vGluT-1 (1:400) and vGluT-2 (1:400); (b) vGluT-1 (1:400) and VAMP-2 (rabbit, 1:300); (c) vGluT-1 (1:400) and SNAP-25A/B (1:800); (d) vGluT-1 (1:400) and syntaxin-1 (1:50); (e) vGluT-2 (1:400) and VAMP-2 (mouse, 1:100); (f) vGluT-2 (1:400) and SNAP-25A/B (1:800); (g) vGluT-2 (1:400) and syntaxin-1 (1:50); (h) GAD-65/67 (mouse, 1:100) and VAMP-2 (rabbit, 1:300); (i) GAD-65/67 (rabbit, 1:100) and SNAP-25A/B (1:800); (j) GAD-65/67 (rabbit, 1:100) and syntaxin-1 (1:50). 4. Incubations for 60 min at RT with the secondary antibodies diluted in PBS: (i) alexa fluor 568 anti-rabbit antibody (goat, 1:100) (Molecular Probes); (ii) alexa fluor 568 anti-mouse antibody (goat, 1:100) (Molecular Probes); (iii) alexa fluor 568 anti-guinea pig antibody (goat, 1:100) (Molecular Probes); (iv) alexa fluor 488 fragment-conjugated anti-rabbit antibody (goat, 1:100) (Molecular Probes, Invitrogen Corporation, Carlsbad, CA, USA); (v) alexa fluor 488 anti-mouse antibody (goat, 1:100) (Molecular Probes); (vi) alexa fluor 488 anti-guinea pig antibody (goat, 1:100) (Molecular Probes); (vii) biotinylated anti-rabbit antibody (donkey, 1:100) (Santa-Cruz Biotechnology Inc., Santa Cruz, CA, USA). 5. Incubation of the sections treated with the biotinylated secondary anti-rabbit antibody for 60 min at RT in the fluorescein-avidin D solution (Vector Laboratories, Burlingame, CA, USA), diluted 5 μg/ml in PBS. 6. Mounting of sections by Vectashield (Vector).

All observations were carried out under the light microscope Axioskop (Zeiss, Germany) equipped with a high-resolution color video camera (Spot Insight, Diagnostic Instruments Inc., MI, USA).

## Results

The patterns of distribution of immunoreactivities for all the markers examined in this study appeared to be comparable in the different lobes and lobules of cerebellum.

### vGluT-1 and vGluT-2, VAMP-2, SNAP-25A/B and syntaxin-1

**Figure 1:**
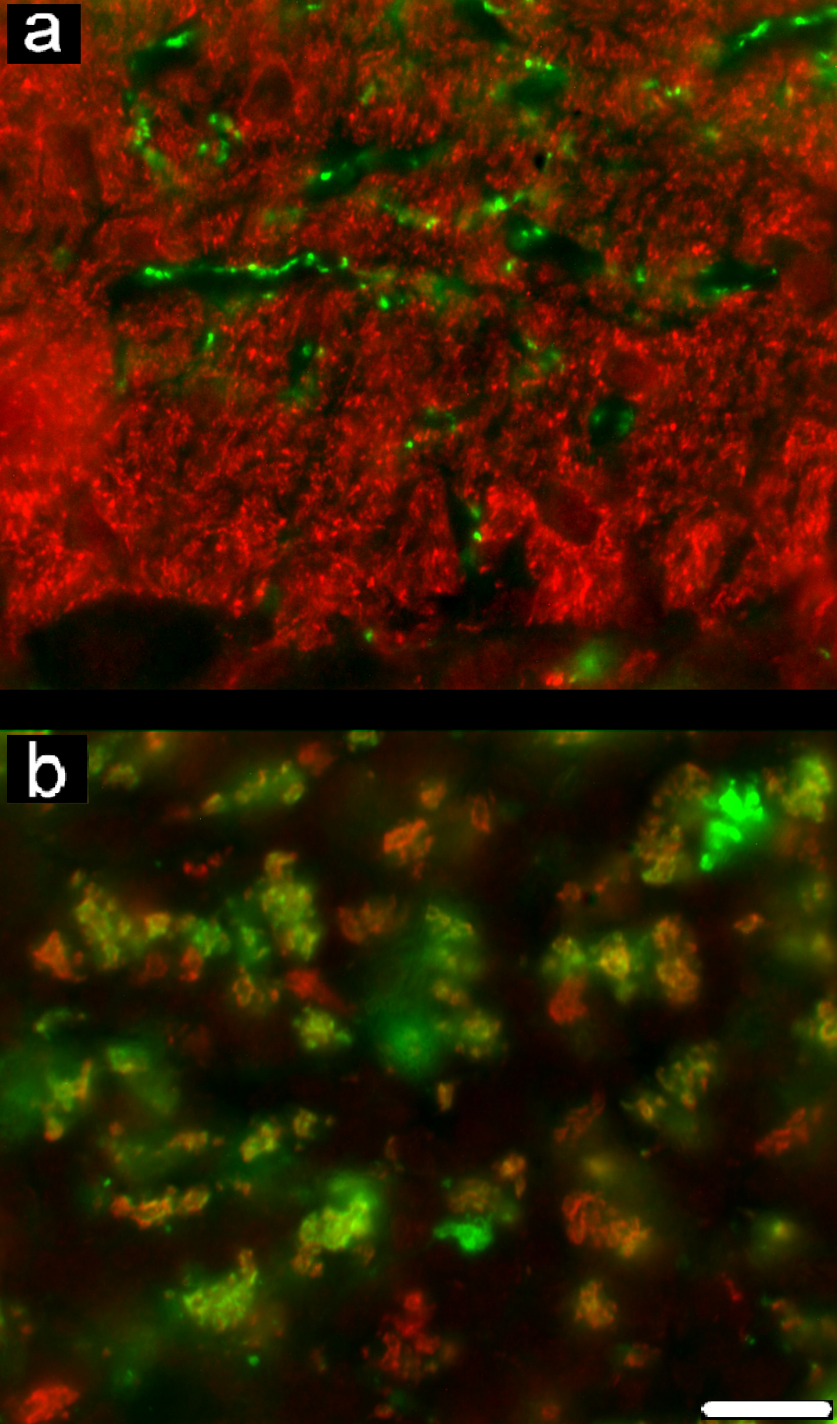
**Double labelling for vGluT-1 and vGluT-2**. (a) In the molecular layer, puncta immulabelled for vGluT-1 (green) are densely distributed in the neuropil; puncta immulabelled for vGluT-2 (red) are localized in the inner zone of the layer, in anatomical relation to body and dendrite trunks of Purkinje neurons. (b) In the granular layer, puncta double immunolabelled for vGluT-1 and vGluT-2 (yellow), and puncta immunoreactive for vGluT-1 or VGluT-2 are present. Scale bar: 10 μm.

**Figure 2:**
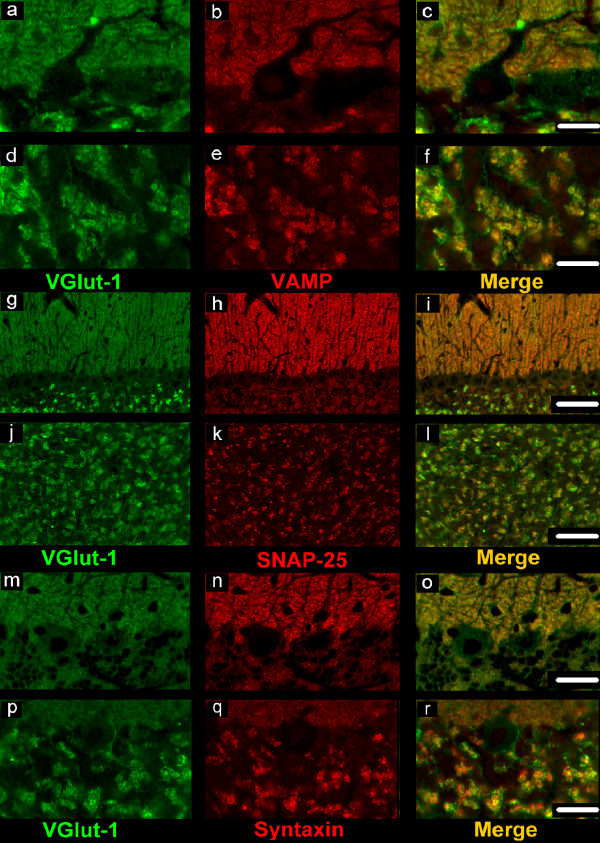
**Double labelling for vGluT-1 and VAMP-2, VGluT-1 and SNAP-25A/B, VGluT-1 and syntaxin-1**. (a, b, c; g, h, i; m, n, o): molecular/Purkinje layer; (d, e, f; j, k, l; p, q, r): granular layer. In the molecular/Purkinje and granular layers, numerous punctate elements which display co-localization of vGluT-1 with VAMP-2 (c, f) or SNAP-25A/B (i, l) or syntaxin-1 (o, r) are seen. However, in all layers, a number of vGluT-1-immunoreactive puncta appear unlabelled for VAMP-2, SNAP-25A/B and syntaxin-1. Scale bars: a, b, c, d, e, f, m, n, o, p, q, r: 20 μm; g, h, i, j, k, l: 50 μm.

**Figure 3:**
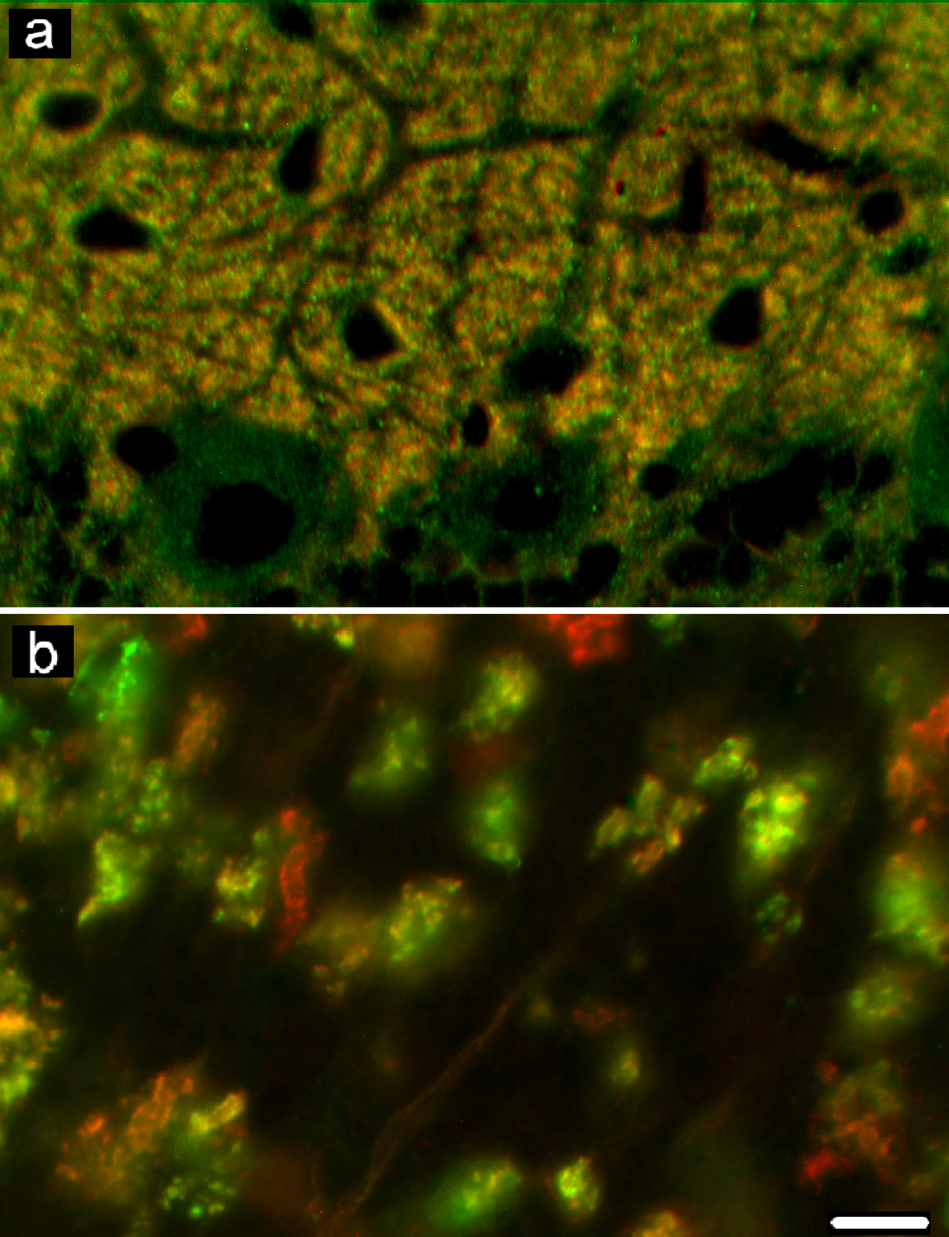
**Double labelling for vGluT-1 and syntaxin-1**. In (a) the molecular layer and (b) granular layer, double labelled punctate elements, putative axon terminals belonging to parallel fibres and mossy fibres, respectively, are seen. In both the layers, subpopulation of terminals positive only for vGluT-1 are present. Scale bar: 10 μm.

**Figure 4:**
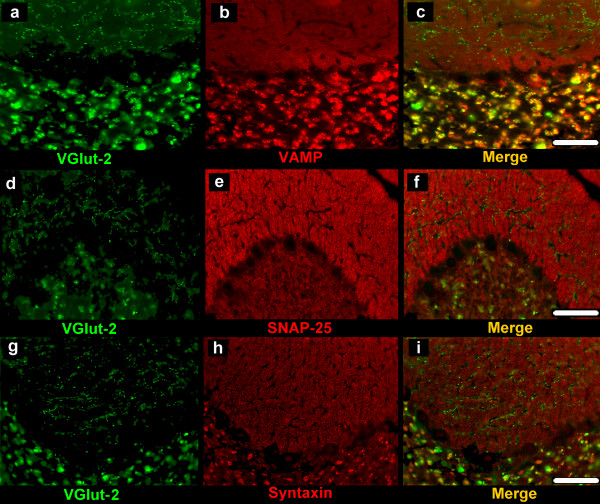
**Double labelling for vGluT-2 and VAMP-2, vGluT-2 and SNAP-25A/B, vGluT-2 and syntaxin-1**. Double immunoreactions reveal absence of co-localization in the molecular/Purkinje layer (c, f, i). vGluT-2-immunoreactive puncta which co-localize VAMP-2, SNAP-25A/B and syntaxin-1 are present in the granular layer (c, f, i). Scale bars: 50 μm.

Immunohistochemical techniques for vGluT-1 and vGluT-2 produced a punctate staining, attributable to the three main systems of glutamatergic terminals in the cerebellar cortex (Figure 1, Figure 2, Figure 3, Figure 4). Precisely, immunohistochemistry for vGluT-1 revealed: in the molecular/Purkinje layer (ML), the terminals of parallel fibres, which were densely and homogeneously distributed in the abundant neuropil interposed among bodies and processes of stellate, basket, Purkinje neurons and blood vessels (Figure 1a); in the granular layer (GL), the terminals of mossy fibres, which were localized in discrete regions of the neuropil comprised among granules (Figure 1b). Immunohistochemistry for vGluT-2 revealed: in the ML, the terminals of climbing fibres, localized in the inner zone of the layer, where they formed discrete rows surrounding body and dendrite trunks of Purkinje neurons (Figure 1a); in the GL, the terminals of mossy fibres, distributed in the neuropil with a pattern similar to that of vGluT-1 immunoreactivity (Figure 1b). Double labelling for vGluT-1 and vGluT-2 revealed co-localization of vGluT-1 and vGluT-2 only in the granular layer, in a subpopulation of terminals of mossy fibres (Figure 1b).

**Figure 5:**
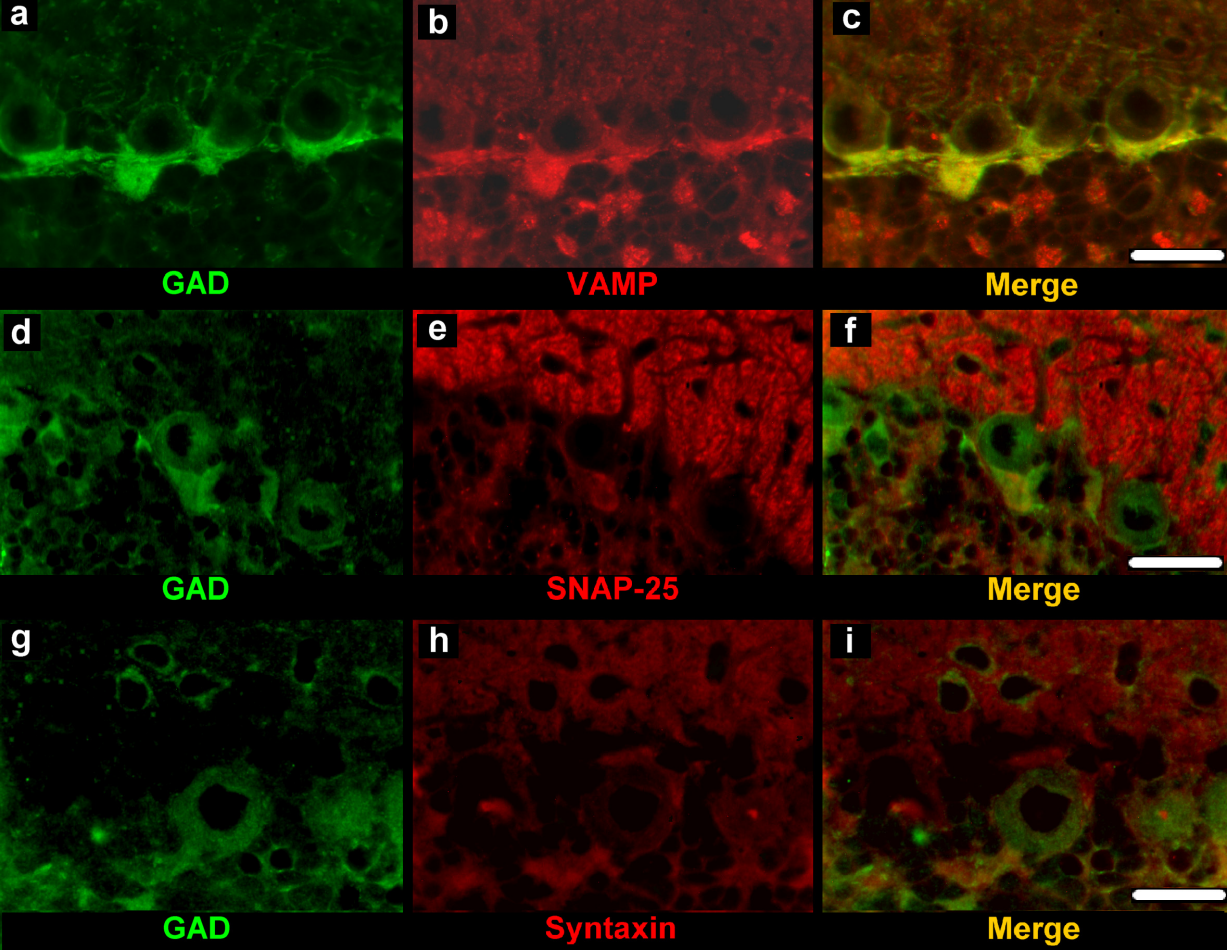
**Double labelling for GAD-65/67 and VAMP-2, GAD-65/67 and SNAP-25A/B, GAD-65/67 and syntaxin-1**. Co-localization is detectable only in puncta localized on the deep pole of the Purkinje neuron body (c, f, i). Scale bars: 20 μm.

**Figure 6:**
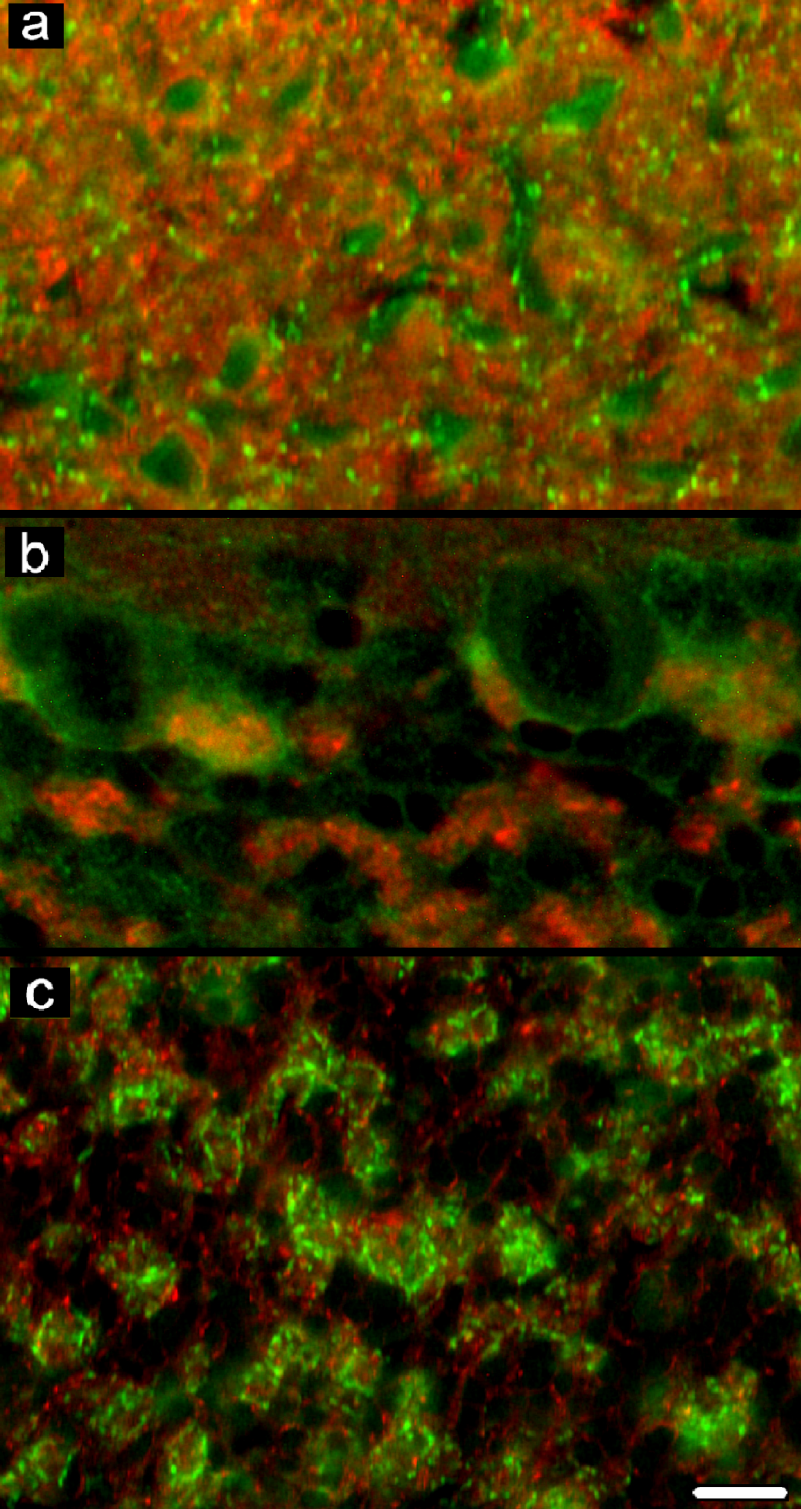
**Double labelling for GAD-65/67 and syntaxin-1**. In (a) the molecular and (b, c) granular layer, puncta co-localizing GAD-65/67 and syntaxin-1 are absent. Puncta which co-localize GAD-65/67 and syntaxin-1 are seen only on the deep pole of Purkinje neuron bodies (b). Scale bar: 10 μm.

Immunoreactions for VAMP-2, SNAP-25A/B and syntaxin-1 produced a diffuse staining in the cortex. The qualitative patterns of distribution of the three examined SNARE were similar. The immunoreactivities were observed in fine, highly concentrated dots, which almost completely filled the neuropil of the whole cortex ('background of positivity'), whereas the neuronal bodies and processes were constantly negative to the immunoreactions (Figure 2, Figure 3, Figure 4, Figure 5, Figure 6).

Double labelling for vGluT-1 and VAMP-2 (Figure 2c, Figure 2f) or SNAP-25A/B (Figure 2i, Figure 2l) or syntaxin-1 (Figure 2o, Figure 2r; Figure 3) revealed numerous punctate elements in the ML and GL, which displayed a co-localization of vGluT-1 with VAMP-2 or with SNAP-25A/B or with syntaxin-1. The double labelled puncta were seen as zones of focal immunoreactivity standing out clearly against the background of positivity provided by the immunoreactivity for the three SNARE proteins. However, in all the layers, a number of vGluT-1-immunoreactive puncta were immunonegative for VAMP-2, SNAP-25A/B and syntaxin-1. Double labelling for vGluT-2 and VAMP-2 (Figure 4c) or SNAP-25A/B (Figure 4f) or syntaxin-1 (Figure 4i) revealed the absence of any co-localization in the ML. Instead, in the GL, a number puncta were observed to co-localize vGluT-2 with the examined SNARE proteins (Figure 4c, Figure 4f, Figure 4i).

### GAD-65/67, VAMP-2, SNAP-25A/B and syntaxin-1

Immunohistochemistry for GAD-65/67 revealed punctate elements, putative GABAergic axon terminals, as well as neuronal bodies and processes, distributed throughout the cortex with layer specific patterns of distribution (Figure 5a, Figure 5d, Figure 5g).

In the double labelling for GAD-65/67 and VAMP-2 (Figure 5c) or SNAP-25A/B (Figure 5f) or syntaxin-1 (Figure 5i; Figure 6), the GAD-65/67-immunoreactive elements appeared as areas of focal positivity against the background of positivity displayed by VAMP-2 or SNAP-25A/B or syntaxin-1 immunoreactivities. Some puncta at the deep pole of the Purkinje neuron body, where are concentrated the axon terminals of basket neurons (pinceau), displayed a co-localization of GAD-65/67 and the SNARE proteins studied (Figure 5c,Figure 5f, Figure 5i; Figure 6b). No co-localization was observed in the GL (Figure 5c, Figure 5f, Figure 5i; Figure 6c).

## Discussion

The demonstration in the rat cerebellar cortex of diffuse immunoreactivity for VAMP (isoform 2), SNAP-25 (isoforms A and B) and syntaxin (isoform 1), morphologically confirms a massive involvement of SNARE mechanisms in the regulation of trafficking of synaptic vesicles and release of neurotransmitters at cortex synapses [11,13,48-51,57]. The SNARE proteins examined were found densely distributed in the neuropil of all layers of the cerebellar cortex. However, the characteristic background of positivity displayed by the proteins indicates that they are not localized exclusively in synaptic terminals, but ubiquitously in neuronal processes until their more distal ramifications. This observation agrees with the assumption that SNARE proteins may be involved also in non-synaptic functions, such as the regulation of morphogenic processes (axon elongation, synapse formation, receptor recycling) [57,66-68]. Moreover, the presence of these proteins in non-synaptic regions could indicate that mechanisms of vesicle exocytosis may occur not only at synapses, but also outside of them, along the whole neuronal membrane. This observation could constitute the morphological basis of an extrasynaptic release of neurotransmitter, which act diffusely within a volume of nervous tissue (volume transmission) rather than at the level of a certain number of spatially separate synapses.

The novelty of this study derives mainly from results obtained by double labelling experiments for the SNARE proteins and markers of glutamatergic (vGluT-1 and vGluT-2) and GABAergic (GAD-65/67) synapses. These experiments allowed us to recognize the glutamatergic or GABAergic axon terminals which contain the examined SNARE proteins or not. In fact, the immunoreactivities for VAMP-2, SNAP-25A/B and syntaxin-1, widely distributed in the cortex, do not provide precise information about the synapses in which they are expressed. On the contrary, the focal immunoreactivity for vGluT-1 and vGluT-2, and for GAD-65/67, owing to their well-defined distributional patterns in the cerebellar cortex, can be useful to recognize the glutamatergic synapses and GABAergic synapses, respectively. The results evidence, among the glutamatergic and GABAergic synapses of the cerebellar cortex, subpopulations which express the SNARE proteins and subpopulations which do not express them.

### Glutamatergic synapses

The glutamatergic synapses of the cerebellar cortex were recognized on the basis of the topographic distribution in the cerebellar cortex layers and expression of vesicular glutamate transporters, vGluT-1 and/or vGluT-2 [39,40,58-62]. In the present study, we observed that the glutamatergic synapses between the parallel fibre terminals and the dendritic tree of Purkinje neurons frequently display co-localization of vGluT-1 with the three SNARE proteins examined. On the contrary, the glutamatergic synapses between the climbing fibre terminals and the body and proximal part of dendrite tree of Purkinje neurons express vGluT-2, but are negative for the SNARE proteins. In the granular layer, most of the glutamatergic terminals of the mossy fibre and of unipolar brush neuron axon appear immunoreactive for vGluT-1 or vGluT-2 and for the three SNARE proteins examined. However, some terminals of parallel fibres (vGluT-1 immunoreactive) and mossy fibres (vGluT-1 and/or vGluT-2 immunoreactive) do not express VAMP-2, SNAP-25A/B and syntaxin-1.

These data agree with the assumption that the SNARE mechanisms largely characterize the excitatory synapses and that glutamate-containing vesicles release their content via SNARE-dependent mechanisms [12,15,33,34]. However, this study demonstrates subpopulations of glutamatergic synapses in the cerebellar cortex that do not contain the examined SNARE proteins. They include virtually all synapses from terminals of climbing fibres, but also some of synapses from parallel and mossy fibres. The terminals positive for vGluT-1 and vGluT-2, but negative for VAMP-2, SNAP-25A/B and syntaxin-1 could be glutamatergic terminals caught in a functional phase which do not express detectable levels of SNARE proteins. Alternatively, they could be cross-sectioned glial processes. In fact, studies have reported that processes of astrocytes may express vesicular glutamate transporters [69,70], but never express SNARE proteins (at least the isoforms examined in this study) [71].

### GABAergic synapses

The GABAergic synapses in the molecular layer, mainly represented by the synapses between the axon terminals of stellate neurons and dendritic tree of Purkinje neurons, express GAD-65/67, but are constantly immunonegative for the three SNARE proteins examined. The GABAergic synapses in the granular layer, mainly represented by the synapses between the axon terminals of Golgi, candelabrum and Lugaro neurons and dendrites of granules, express GAD-65/67, but do not express immunoreactivity for the examined SNARE proteins. The only elements which co-localize GAD-65/67 with VAMP-2 or SNAP-25A/B or syntaxin-1 are represented by some axon terminals of basket neurons, making synapse on the deep pole of the Purkinje neurons body.

These results indicate that, in most GABAergic synapses of the cerebellar cortex, VAMP-2, SNAP-25A/B and syntaxin-1 do not participate in SNARE complexes responsible for trafficking of GABA-containing vesicles. This observation largely agrees with the assumption that SNAP-25 independent exocytotic pathways exist to support neurotransmission in GABAergic synapses [12,33], even if this assumption is not accepted by some researchers [14]. Moreover, it should again pointed out that some GABAergic axon terminals of basket neurons are exceptions to this assumption.

## Conclusions

VAMP-2, SNAP-25A/B and syntaxin-1 are differently patterns distributed among the glutamatergic and GABAergic synapses of the adult rat cerebellar cortex. They largely characterize glutamatergic synapses, but lack in subpopulations of them, and are absent in most of GABAergic synapses, with the exception of some synapses between basket and Purkinje neurons.

In the subpopulations of glutamatergic and GABAergic synapses that do not express VAMP-2, SNAP-25A/B and syntaxin-1, alternative mechanisms regulating the exocytosis of glutamate or GABA neurotransmitters may be hypothesized, possibly based on the presence of homologous proteins. The SNARE mechanisms should be present in cerebellar cortex synapses, but in the synapse subpopulations that lack VAMP-2, SNAP-25A/B and syntaxin-1, they should be supported by other proteins, which are able to replace the missing ones, even though they were not labelled by the antibodies used in this study. This hypothesis is supported by the demonstration in the mammalian cerebellar cortex of a number of isoform and homologous proteins different from VAMP-2, SNAP-25A/B and syntaxin-1 (e.g., VAMP-1; SNAP-23, SNAP-47; syntaxin-3A, B, C, D) [13,21,24,34].

Since the presence, or the absence, of co-localizations of markers of glutamate or GABA with the key SNARE proteins examined in this study must be unequivocally established, the present results need to be integrated by further qualitative and quantitative studies carried out using confocal and electron microscopy. The elucidation of the molecular features of the glutamatergic and GABAergic synapses in the cerebellar cortex, and in particular of the mechanisms underlying the interactions between vesicle membrane and presynaptic membrane in these synapses, will be important not only to obtain further insights on the chemical and morphofunctional organization of cerebellar cortex synapses, but also for possible impact in clinical medicine (e.g., for the identification of potential targets for drug therapies that may act selectively at cerebellar glutamatergic or GABAergic synapses).

## Authors' contributions

VB and LL equally contributed to this study, designed the study, carried out experiments, collected data, draft the manuscript. PF carried out experiments, collected data. FG, AR, LB, RC analyzed and discussed the results. BN and DR analyzed and discussed the results, revised the manuscript. GA designed the study, co-ordinated the laboratory activities, analyzed and discussed the results, revised the manuscript.

All authors read and approved the final manuscript. The authors declare that they have no competing interests.
